# Supplementary material for: Classification with a Deferral Option and Low-Trust Filtering for Automated Seizure Detection
Source: Sensors (Basel). 2021 Feb 4;21(4):1046. doi: 10.3390/s21041046 (PMC7913713; doi:10.3390/s21041046)
Supplement: Supplementary file 1 [file sensors-21-01046-s001.pdf]

Supplementary Materials: Classification with a Deferral Option and Low-Trust Filtering for Automated Seizure Detection

Thijs Becker, Kaat Vandecasteele, Christos Chatzichristos, Wim Van Paesschen, Dirk Valkenborg, Sabine Van Huffel and Maarten De Vos

1. Choice of Classification Rule for Low-Trust Filtering

We first tried two other classification rules before settling on the rule used in the main article. We shortly discuss our thought process here. We present results for the CI SVM and trust scores from a trust model trained on the FS labels.

The first classification rule we tried was the following. If more than 0 samples remain after filtering, take the average of the remaining samples. If this average is larger than 0.7, classify the segment as a seizure. If no samples remain after filtering, consider the four highest-trust samples. If three or four of these are predicted as a seizure, classify the segment as a seizure. The resulting behaviour for different filtering percentages is shown in Figure S1. We did not perform cross-validation, but just calculated the average performance metrics on all patients. We see a clear optimum around 2%, which was also found with the nested cross-validation approach from the article. This classification rule has two disadvantages. Firstly, it is discontinuous in the number of samples that is considered: there is a jump from one to four considered samples after the last sample is filtered. Secondly, the FDR (called FP rate in Figure S1) increases significantly when a large number of segments is filtered.

The second classification rule we tried was the following. If more than eight samples remain after filtering, take the average of the remaining samples. If this average is larger than 0.7, classify the segment as a seizure. If between one and eight samples remain after filtering, classify the segment as a seizure if the average of the remaining samples is equal to 1.0. If no samples remain after filtering, consider the six highest-trust samples. If all of these are predicted as a seizure, classify the segment as a seizure. The resulting behaviour for different filtering percentages is shown in Figure S2. The optimum stays around 2%, but the FDR always stays below the FDR at 0% filtering. However, the rule is still discontinuous in the number of samples that is considered.

The final rule, our third attempt, is described in the main article. At least five segments are always considered, so the rule is continuous in the number of samples that is considered. There is also no arbitrary threshold in the rule: we keep the 0.7 threshold of the original classification rule, regardless of the number of remaining segments.

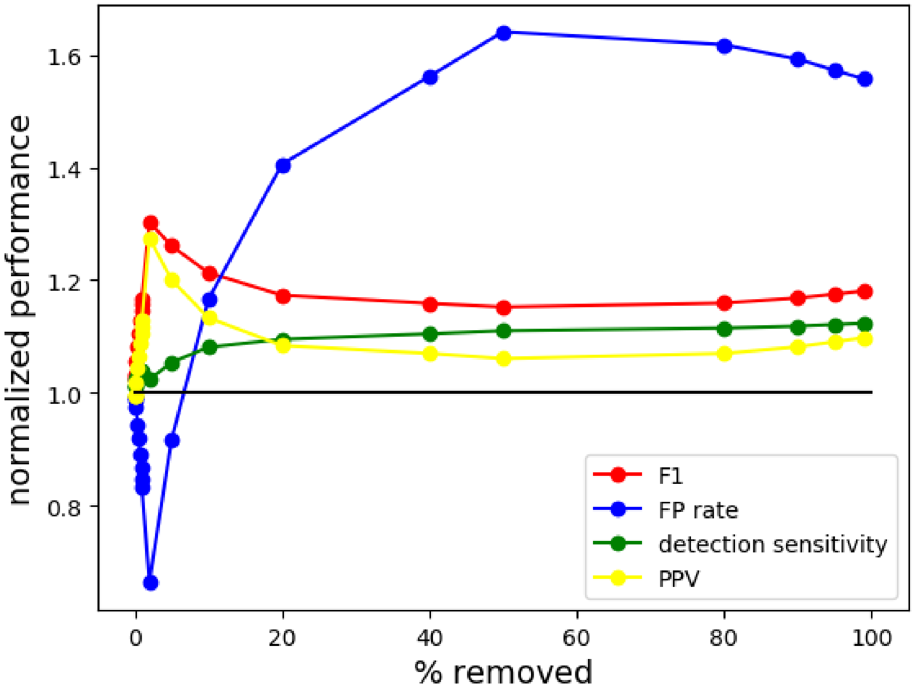

Figure S1. Average performance on all patients (no cross-validation) as a function of the percent of 2-second segments that are filtered (% removed), for the first rule investigated. Performance is normalized with respect to the performance when there is no filtering. Note that FDR is called FP rate.

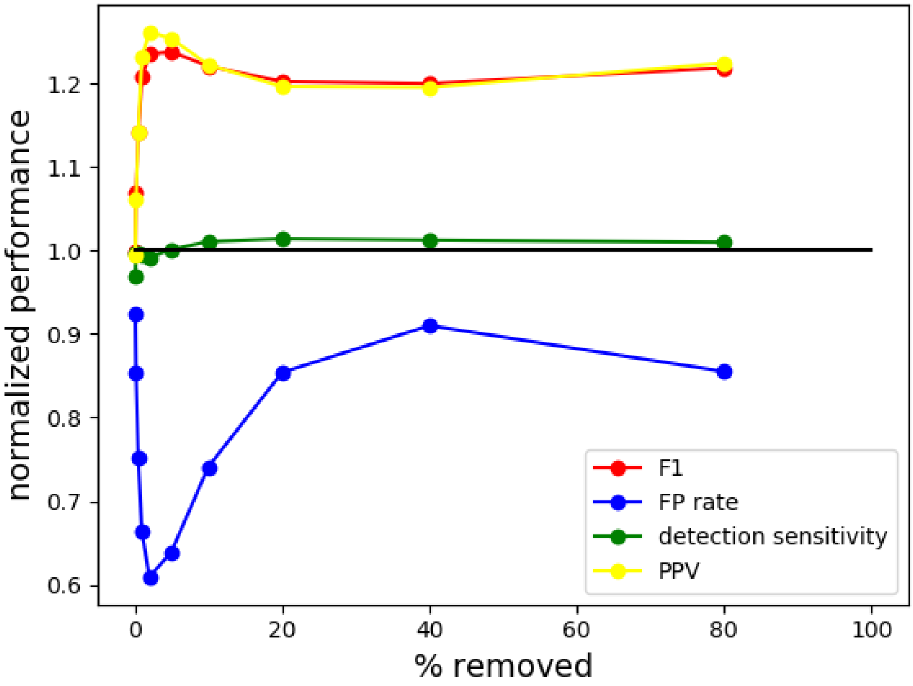

Figure S2. Average performance on all patients (no cross-validation) as a function of the percent of 2-second segments that are filtered (% removed), for the second rule investigated. Performance is normalized with respect to the performance when there is no filtering. Note that FDR is called FP rate.

2. Classification With A Deferral Option: Extra Results

The results for the performance as a function of the fraction of deferred data with trust models trained on the CI labels are shown for the CI SVM in Figure S3 and for the FS SVM in Figure S4. Similar behaviour was observed compared to the trust models trained on the FS labels, which are shown in the main article.

The number and average length of the deferred segments as functions of the fraction of deferred data are plotted for the FS SVM in Figure S5.

In Figure S6 we plot the FDR as a function of the fraction of the data that is deferred to a human annotator, for different p_{low}. In contrast to our main approach, the segments that contain seizure flags are not automatically the first to be deferred. If a seizure flag is in a deferred segment for at least one second, we assume that it is completely checked by the human annotator, even if a part is checked by the algorithm. We observe that the optimal p_{low} is 5%. This is in agreement with our conclusion for the optimal p_{low} for the detection sensitivities, as discussed in the main article.

In the main article, we deferred the same percentage of segments for each patient. We also investigated a strategy where a different percentage of segments is deferred per patient. The numerical threshold at which to defer a segment is the average of the optimal thresholds of the two validation sets. The comparison between these two strategies for the CI SVM with SVM confidences is shown in Figure S7. The strategy where the same percentage of segments is removed is clearly superior. Similar curves are obtained for other models (FS SVM) and confidence measures (trust models trained on the CI or FS labels).

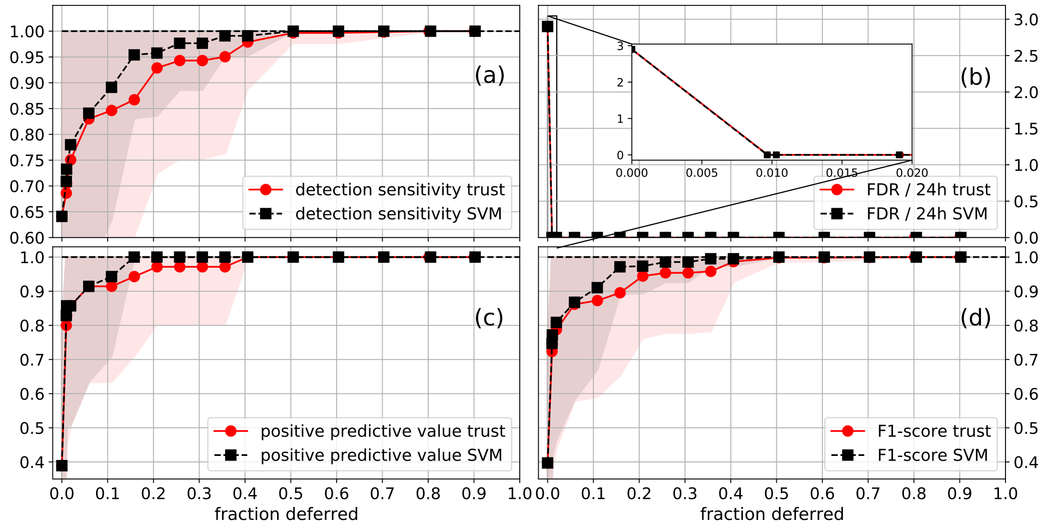

Figure S3. Average (**a**) detection sensitivity; (**b**) FDR/24h; (**c**) PPV; (**d**) F1-score as a function of the fraction of the data that is deferred to a human annotator, for the CI SVM ($p_{\text{low}} = 5$). The standard deviation of the performance is shown as a shaded area, with the upper values capped at one. Segments are deferred using the SVM confidences (SVM) or trust scores (trust) from a trust model trained on the CI labels. The first point with fraction deferred > 0 is the performance when all segments that contain a seizure flag are deferred. The inset plotted in the FDR figure shows that around 1% of the EEG data is contained in segments that contain seizure flags.

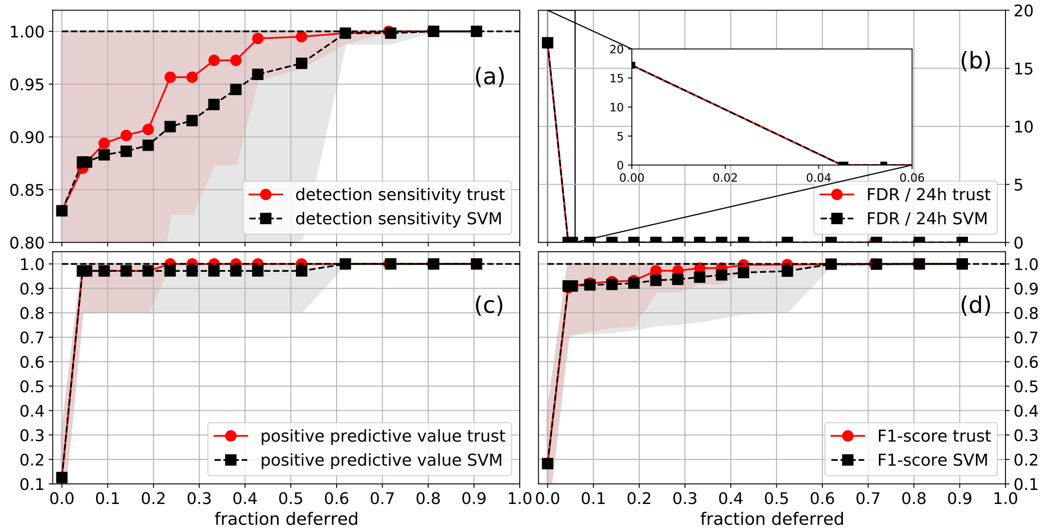

Figure S4. Average (**a**) detection sensitivity; (**b**) FDR/24h; (**c**) PPV; (**d**) F1-score as a function of the fraction of the data that is deferred to a human annotator, for the FS SVM ($p_{\text{low}} = 5$). The standard deviation of the performance is shown as a shaded area, with the upper values capped at one. Segments are deferred using the SVM confidences (SVM) or trust scores (trust) from a trust model trained on the CI labels. The first point with fraction deferred > 0 is the performance when all segments that contain a seizure flag are deferred. The inset plotted in the FDR figure shows that around 4.5% of the EEG data is contained in segments that contain seizure flags.

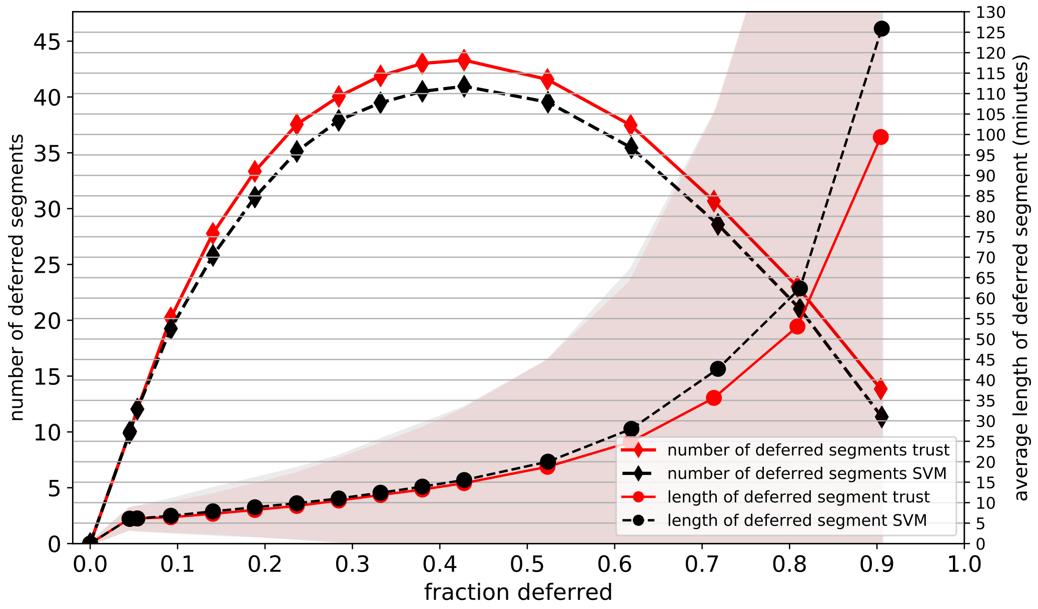

Figure S5. Average number and average length (minutes) of the deferred segments, as functions of the fraction of the data that is deferred to a human annotator, for the FS SVM ($p_{\text{low}} = 5$). The standard deviation of the length is shown as a shaded area, with the lower values capped at zero. Segments are deferred using the SVM confidences (SVM) and trust scores (trust) from a trust model trained on the FS labels.

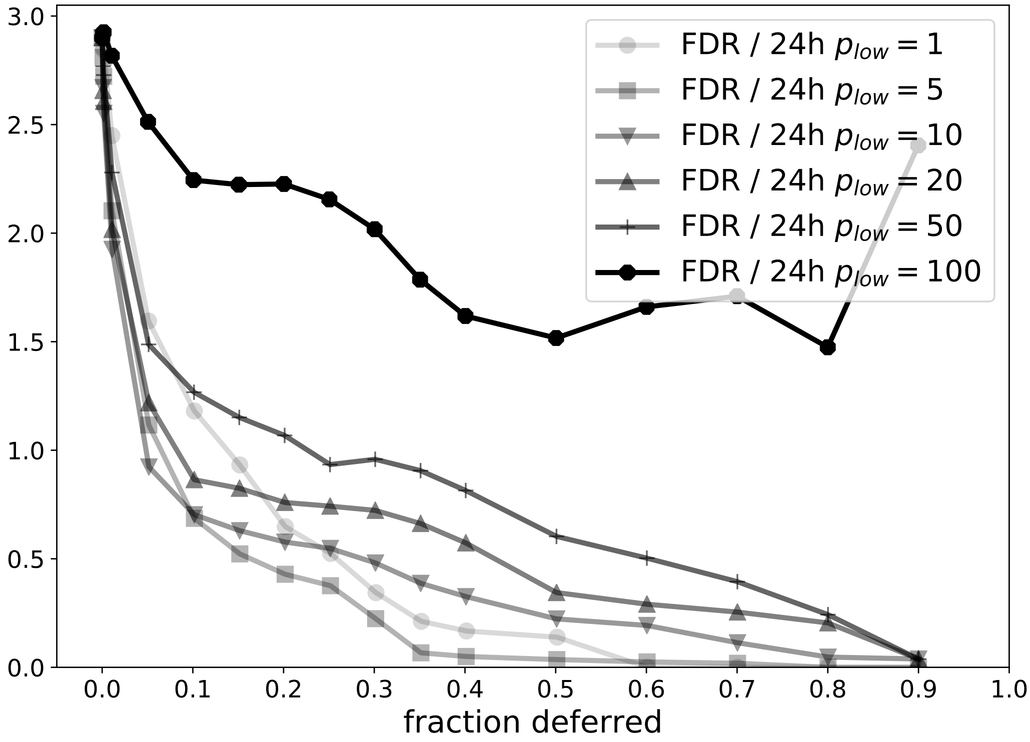

Figure S6. Average FDR as a function of the fraction of the data that is deferred to a human annotator, for the CI SVM, for different p_{low}. The segments that contain seizure flags are not deferred at the start. The trust scores are obtained from a model trained on the FS labels.

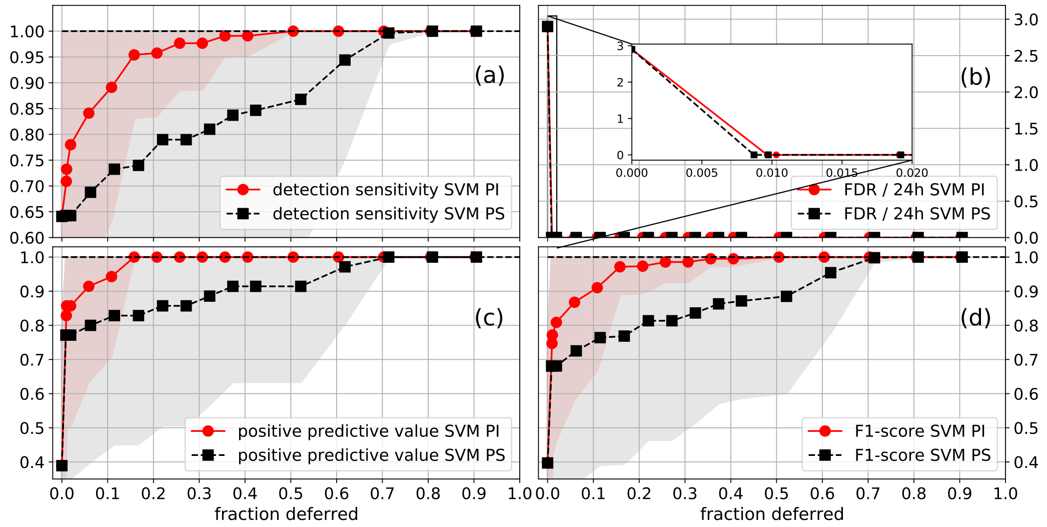

Figure S7. Average (**a**) detection sensitivity; (**b**) FDR/24h; (**c**) PPV; (**d**) F1-score as a function of the fraction of the data that is deferred to a human annotator, for the CI SVM ($p_{\text{low}} = 5$). The standard deviation of the performance is shown as a shaded area, with the upper values capped at one. Segments are deferred using the SVM confidences. The first point with fraction deferred > 0 is the performance when all segments that contain a seizure flag are deferred. The inset plotted in the FDR figure shows that around 1% of the EEG data is contained in segments that contain seizure flags. In one strategy we defer the same percentage of segments per patient, referred to as patient-independent (PI). In the other strategy we defer a different percentage per patient, referred to here as patient-specific (PS). The PI strategy is the one from the main article.

3. Low-Trust Filtering: Extra Results

Classifying with a defer option, with and without starting with a model on which LTF has been performed, is shown in Figure S8 for the CI SVM and in Figure S9 for the FS SVM. Although the performance at 0% deferral is better, there is no real advantage for larger deferral percentages. This is most likely because the additionally detected seizures from LTF are in segments with low confidence, since those are the segments that are expected to benefit from LTF. These segments are among the first to be deferred. The percentage that needs to be deferred to obtain a FDR of 0 is similar, and even slightly lower without LTF for the CI SVM. Although one generally expects the reverse behaviour, as can be seen for the FS SVM, the fact that false positives are clustered combined with our deferral strategy can lead to this behaviour.

Figure S10 shows a visualisation of a new seizure detection after performing LTF.

We also investigated a strategy of LTF where a different percentage of lowest-trust segments is filtered per patient. The numerical threshold at which to filter is determined from the average of the optimal threshold of the two validation folds. We call this the patient-independent (PI) strategy (same percentage filtered per patient) and patient-specific (PS) strategy (different percentage filtered per patient). There is no big difference between the strategies, as can be seen for the CI SVM with LTF from trust scores from trust models trained on the FS labels (Table 1) or the CI labels (Table 2), and the SVM confidences (Table 3).

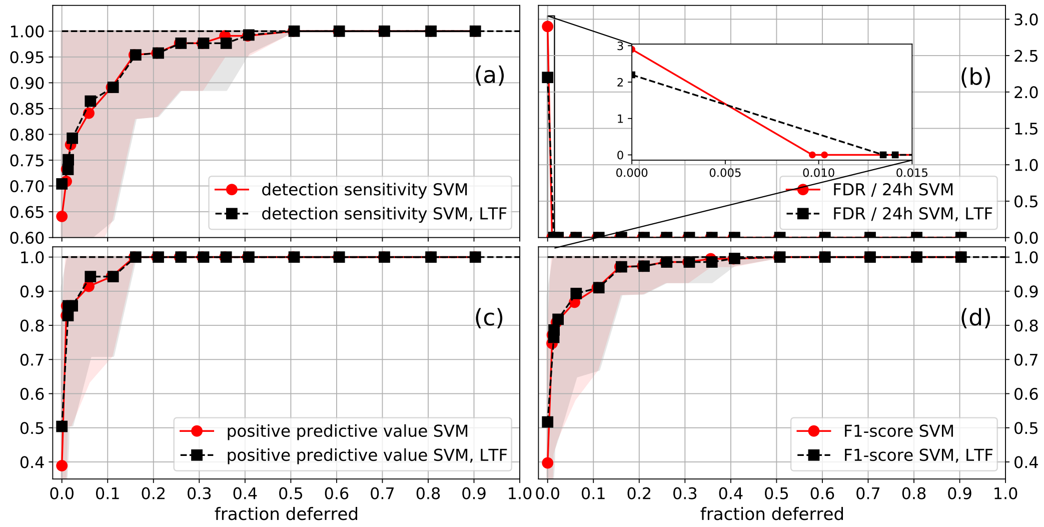

Figure S8. Average (**a**) detection sensitivity; (**b**) FDR/24h; (**c**) PPV; (**d**) F1-score as a function of the fraction of the data that is deferred to a human annotator, for the CI SVM ($p_{\text{low}} = 5$). The standard deviation of the performance is shown as a shaded area, with the upper values capped at one. Segments are deferred using the SVM confidences (SVM). The first point with fraction deferred > 0 is the performance when all segments that contain a seizure flag are deferred. Low-trust filtering (LTF) is either performed or not.

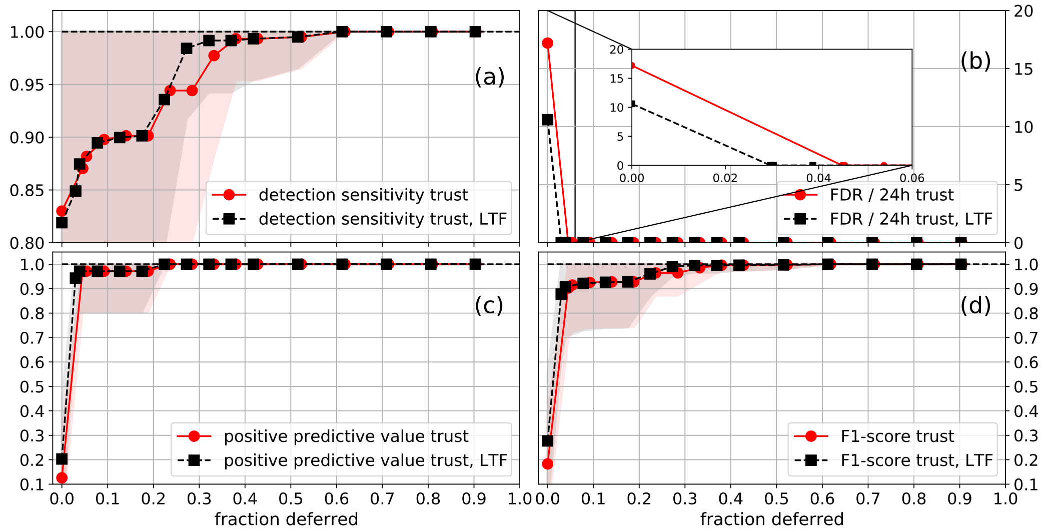

Figure S9. Average (**a**) detection sensitivity; (**b**) FDR/24h; (**c**) PPV; (**d**) F1-score as a function of the fraction of the data that is deferred to a human annotator, for the FS SVM ($p_{\text{low}} = 5$). The standard deviation of the performance is shown as a shaded area, with the upper values capped at one. Segments are deferred using trust scores (trust) from a trust model trained on the CI labels. The first point with fraction deferred > 0 is the performance when all segments that contain a seizure flag are deferred. Low-trust filtering (LTF) is either performed or not.

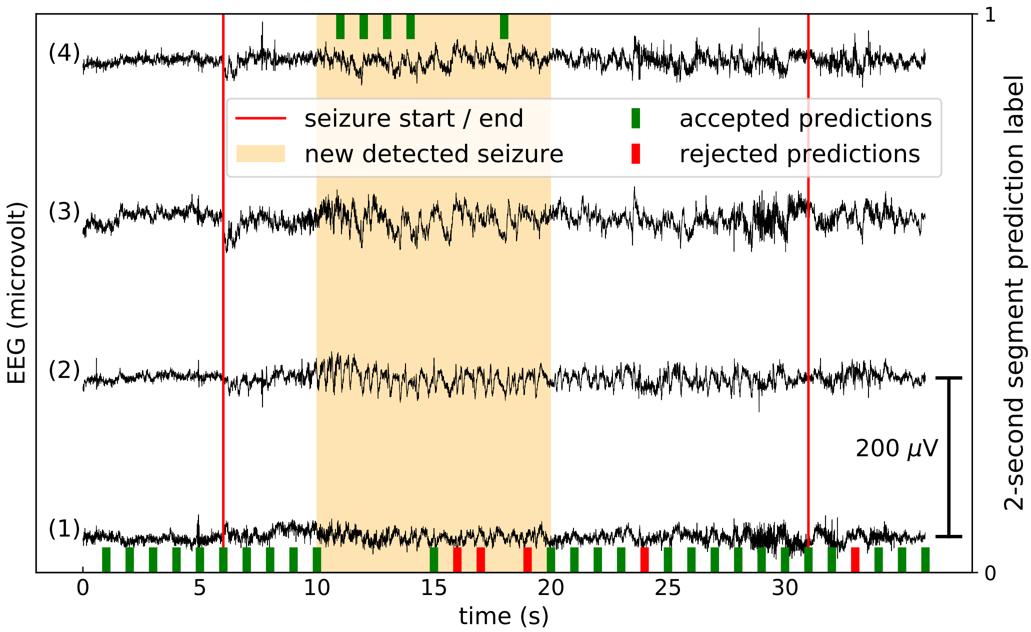

Figure S10. A visualisation of a new detection after low-trust filtering for the CI SVM. (1) Crosshead 1, (2) crosshead 2, (3) unilateral left, (4) unilateral right. If the SVM predicts 0, a bar on the x-axis in the middle of the corresponding 2-second segment is shown. If the SVM predicts 1, a bar on top of the figure is shown. Predictions that are flagged as untrustworthy are shown as red bars; otherwise they are green. The new seizure flag is shown in orange.

Table 1: Mean (standard deviation) performances of: CI SVM and LTF with trust models trained on the FS (trust FS) labels. Either the same percentage is filtered per patient (patient-independent (PI)) or a different percentage is filtered per patient (patient-specific (PS)). A statistically significant improvement or degradation in performance compared to the original SVM is denoted by a star. The best result (or not significantly different from the best result) is shown in bold.

**Metric \ Method**	**CI SVM**	**LTF, Trust FS, PI**	**LTF, Trust FS, PS**
DS (%)	64.1 (41.5)	**71.4*** (**38.6**)	**70.8** (**38.9**)
FDR / 24h	2.9 (5.6)	**2.3*** (**4.7**)	**2.2*** (**4.2**)
PPV (%)	38.9 (38.9)	**50.8*** (**40.6**)	**49.2*** (**40.3**)
F1-score (%)	39.7 (34.2)	**52.1*** (**36.5**)	**50.9*** (**36.4**)
detection delay (s)	22.1 (13.2)	**21.4** (**12.0**)	**21.5** (**12.0**)

Table 2: Mean (standard deviation) performances of: CI SVM and LTF with trust models trained on the CI (trust CI) labels. Either the same percentage is filtered per patient (PI) or a different percentage is filtered per patient (PS). A statistically significant improvement or degradation in performance compared to the original SVM is denoted by a star. The best result (or not significantly different from the best result) is shown in bold.

**Metric \ Method**	**CI SVM**	**LTF, Trust CI, PI**	**LTF, Trust CI, PS**
DS (%)	**64.1** (**41.5**)	**63.8** (**41.0**)	**63.8** (**41.0**)
FDR / 24h	2.9 (5.6)	**1.7*** (**3.8**)	**1.5*** (**3.2**)
PPV (%)	38.9 (38.9)	**48.6*** (**40.5**)	**48.7*** (**40.5**)
F1 (%)	39.7 (34.2)	**49.2*** (**36.9**)	**49.2*** (**36.9**)
detection delay (s)	**22.1** (**13.2**)	23.2* (12.2)	23.2* (12.2)

Table 3: Mean (standard deviation) performances of: CI SVM and LTF with the confidences of this SVM (CI SVM conf.). Either the same percentage is filtered per patient (PI) or a different percentage is filtered per patient (PS) A statistically significant improvement or degradation in performance compared to the original SVM is denoted by a star. The best result (or not significantly different from the best result) is shown in bold.

**Metric \ Method**	**CI SVM**	**CI SVM conf., PI**	**CI SVM conf., PS**
DS (%)	**64.1** (**41.5**)	**64.1** (**41.5**)	**64.1** (**41.5**)
FDR / 24h	**2.9** (**5.6**)	5.4* (11.7)	5.4* (11.8)
PPV (%)	**38.9** (**38.9**)	38.7* (39.0)	38.5 (39.1)
F1 (%)	**39.7** (**34.2**)	39.4* (34.3)	39.1 (34.2)
detection delay (s)	22.1 (13.2)	**21.9*** (**13.0**)	22.1 (13.2)

Abbreviations

The following abbreviations are used in this manuscript:

CI	clear ictal
EEG	electroencephalographic
FDR	false detection rate
FP	false positive
FS	full seizure
LTF	low-trust filtering
PPV	positive predictive value
PI	patient independent
PS	patient specific
SVM	support vector machine
